# Genome-wide association with footrot in hair and wool sheep

**DOI:** 10.3389/fgene.2023.1297444

**Published:** 2024-01-15

**Authors:** Mehmet Ulas Cinar, Ryan D. Oliveira, Tracy S. Hadfield, Anne Lichtenwalner, Richard J. Brzozowski, C. Thomas Settlemire, Susan G. Schoenian, Charles Parker, Holly L. Neibergs, Noelle E. Cockett, Stephen N. White

**Affiliations:** ^1^ Department of Veterinary Microbiology and Pathology, Washington State University, Pullman, WA, United States; ^2^ Department of Animal Science, Faculty of Agriculture, Erciyes University, Kayseri, Turkiye; ^3^ Department of Animal, Agricultural Experiment Station, Dairy and Veterinary Sciences, Utah State University, Logan, UT, United States; ^4^ School of Food and Agriculture, University of Maine, Orono, ME, United States; ^5^ Cooperative Extension, University of Maine, Orono, ME, United States; ^6^ Biology Department, Bowdoin College, Brunswick, ME, United States; ^7^ Western Maryland Research and Education Center, University of Maryland, College Park, MD, United States; ^8^ Department of Animal Sciences, Professor Emeritus, The Ohio State University, Columbus, OH, United States; ^9^ Department of Animal Science, Washington State University, Pullman, WA, United States; ^10^ Animal Disease Research Unit, Agricultural Research Service, U.S. Department of Agriculture, Pullman, WA, United States; ^11^ Center for Reproductive Biology, Washington State University, Pullman, WA, United States

**Keywords:** GWAS, SNP, genetic background, *Ovis aries*, hoof health

## Abstract

Ovine footrot is an infectious disease with important contributions from *Dichelobacter nodosus* and *Fusobacterium necrophorum*. Footrot is characterized by separation of the hoof from underlying tissue, and this causes severe lameness that negatively impacts animal wellbeing, growth, and profitability. Large economic losses result from lost production as well as treatment costs, and improved genetic tools to address footrot are a valuable long-term goal. Prior genetic studies had examined European wool sheep, but hair sheep breeds such as Katahdin and Blackbelly have been reported to have increased resistance to footrot, as well as to intestinal parasites. Thus, footrot condition scores were collected from 251 U.S. sheep including Katahdin, Blackbelly, and European-influenced crossbred sheep with direct and imputed genotypes at OvineHD array (>500,000 single nucleotide polymorphism) density. Genome-wide association was performed with a mixed model accounting for farm and principal components derived from animal genotypes, as well as a random term for the genomic relationship matrix. We identified three genome-wide significant associations, including SNPs in or near *GBP6* and *TCHH*. We also identified 33 additional associated SNPs with genome-wide suggestive evidence, including a cluster of 6 SNPs in a peak near the genome-wide significance threshold located near the glutamine transporter gene *SLC38A1*. These findings suggest genetic susceptibility to footrot may be influenced by genes involved in divergent biological processes such as immune responses, nutrient availability, and hoof growth and integrity. This is the first genome-wide study to investigate susceptibility to footrot by including hair sheep and also the first study of any kind to identify multiple genome-wide significant associations with ovine footrot. These results provide a foundation for developing genetic tests for marker-assisted selection to improve resistance to ovine footrot once additional steps like fine mapping and validation are complete.

## 1 Introduction

Footrot, or infectious pododermatitis, is a hoof infection commonly found in sheep, goats, and cattle. Although footrot was first described more than 180 years ago, it is a complex disease still endemic in many countries (Zanolari et al., 2021). Ovine footrot is caused by an interaction of two anaerobic, Gram-negative bacteria: *D. nodosus* (formerly *Bacteroides nodosus*) and *F. necrophorum* (formerly *Sphaerophorus necrophorus*). However, *Dichelobacter nodosus* is the primary causative agent of footrot in sheep (Kennan et al., 2014). Initial colonization of the hoof by opportunistic bacteria, including the ruminant digestive tract commensal bacterium *Fusobacterium necrophorum*, is followed by infection with *D. nodosus*, and interaction between these two pathogens causes footrot in sheep. Ovine footrot is characterized by the separation of the keratinous hoof from the underlying tissue and causes severe lameness (Kennan et al., 2011). The annual costs of footrot were estimated at £24.4 million in UK (Nieuwhof and Bishop, 2005) and $18.4 M in Australia (Sackett et al., 2007; Smith et al., 2022), which corresponds to £1.32 and £0.15 per living ewe and lamb, respectively (Nieuwhof and Bishop, 2005). In Switzerland, annual costs for footrot were estimated at CHF33 million for the sheep population (Zingg et al., 2017). Affected sheep frequently experience pain, discomfort, and reduced mobility which affects their ability to access feed (Abbott and Lewis, 2005). Thus, it is not surprising that affected sheep can experience reduced growth rates and wool production. For instance, one study showed that lambs with footrot reached slaughter weight 31.9 days later than lambs without footrot (Zingg et al., 2017).

Currently, a variety of different footrot management and treatment approaches are utilized world-wide. These include foot trimming, foot baths/foot soaks with zinc sulfate and copper sulfate, injection of antibiotics (penicillin and streptomycin combinations), and topical medications or vaccination against *D. nodosus*. None of these interventions is perfect, and the best results are obtained when several methods are combined (Bennett and Hickford, 2011). Variation in management and treatment reflects variation in stocking rate (of importance with a contagious disease), the size of flocks, the cost of labor for labor-intensive management practices, and the cost and availability of remedies and acceptability of the various management and treatment regimes in different markets. There remains a need for additional minimally labor-intensive tools to reduce both losses and treatment costs of ovine footrot.

Since pathogen persistence in the environment depends on the host (Clifton et al., 2019), one possibility is to use genetic resistance to footrot as a prevention tool. Demonstration of genetic variance can provide a sense of the promise of such a strategy, and studies dissecting genetic variation involved in degrees of footrot resistance have been ongoing for the last 4 decades (Escayg et al., 1997). Moderate heritability has been estimated for ovine footrot, generally between 0.20 and 0.30 depending on breed and phenotypic scoring method (Emery et al., 1984; Raadsma et al., 1994; Nieuwhof et al., 2008a; Raadsma and Conington, 2011), demonstrating that footrot resistance is a heritable trait. Breed differences have been observed, including that Merino sheep are particularly susceptible to footrot, while others such as Romney are more resistant (Emery et al., 1984). These results suggest there is potential for development of genetic tools to improve footrot resistance, as simple phenotypic selection has led to long-term genetic improvement (Parker et al., 1983; Conington et al., 2008).

To enhance genetic gains, the identification of specific genes and molecular markers associated with footrot resistance is needed. Although a few genetic markers for natural resistance to footrot have been identified (Litchfield et al., 1993; Escayg et al., 1997; Niggeler et al., 2017), there is still a paucity of information about genetic variation in susceptibility to ovine footrot. The role of the major histocompatibility complex (MHC) in modulating immune responses, and subsequently disease susceptibility for both the Class I and Class II regions, has been investigated in relation to footrot resistance (Litchfield et al., 1993; Escayg et al., 1997; Hickford et al., 2004; Raadsma and Dhungyel, 2013). For genome wide association studies, only two studies (Mucha et al., 2015; Niggeler et al., 2017), have been reported and they were focused solely on European wool sheep breeds. Katahdins and other hair sheep show distinct genetic heritage (Spangler et al., 2017) which has manifested in demonstrated differences in disease resistance traits between hair and wool sheep (Vanimisetti et al., 2004). Some have suggested that hair breeds like Katahdin and Blackbelly might be more resistant to footrot than other sheep (Yazwinski et al., 1979; Zajac, 1995; Bishop and Morris, 2007; Azarpajouh, 2014; de Almeida, 2018), and there has not been an investigation of the genetics of ovine footrot susceptibility at the genome-wide level in these breeds. Therefore, the main aim of this study was to undertake a genome-wide association study (GWAS) to investigate Single Nucleotide Polymorphisms (SNPs) and identify genes linked to footrot susceptibility in North American hair and wool sheep.

## 2 Materials and methods

### 2.1 Animals and phenotyping

Over a 4-year period (2010–14), as part of a NE-SARE-funded study to teach producers a method for elimination of footrot on NE sheep farms, the research team visited sheep farms at least twice in 6 northeastern states (Maine, New Hampshire, Vermont, Pennsylvania, Maryland and New York). All farms had self-identified as affected with ovine footrot. As part of a 4-week, multi-visit farm protocol for footrot control, the research team inspected and trimmed sheep hooves on the initial farm visit. The research team categorized each sheep as being free of any signs of footrot (score 1), showing signs suggestive of footrot (odor, interdigital inflammation; score 2) or having overt footrot (keratin lesions such as undermining of the sole, odor; score 3). Additional details on the scoring system may be found in Supplementary Figure S1. For each sheep, the highest score for any individual hoof was taken as the final score for the animal. In addition to the foot score for each sheep, breed, or breed group (e.g., for crossbreds) was recorded. All farms had one or more sheep with footrot, indicating the presence of the etiologic agents on the farm’s property. Farms with prevalence of 10% or above in the flock were selected for inclusion in the study. Production systems varied in size and breed composition, including fraction of crossbred sheep. Sheep age was not available for every flock, but average age was approximately 2 years for those where it was recorded. Anticoagulated (EDTA) blood was collected once during the study from each sheep for DNA extraction and further analysis. The dataset consisted of 251 sheep from 9 farms including Katahdin and Blackbelly hair sheep, Merino, Polypay, and other wool sheep from European-influenced breeds, plus crossbred sheep.

### 2.2 Genotypes and imputation

Genomic DNA was extracted as previously described (White et al., 2012). Briefly, DNA was isolated using the Invitrogen GeneCatcher™ gDNA 3–10 mL Blood Kit as per manufacturers' instructions (Life Technologies, Carlsbad, CA, US). DNA samples were checked for quality and quantity using an ND-1000 spectrophotometer (Nanodrop, Wilmington, DE, US) and equilibrated to 50 ng/μL for genotyping. Animals were genotyped in the GeneSeek laboratory (Lincoln, NE, United States) using the Illumina ovine HD (Kijas et al., 2014) and the Illumina ovine SNP50 BeadChips (Becker et al., 2010) (Illumina Inc., San Diego, CA, United States). For 200 sheep, genotypes were collected with the Illumina OvineHD array. An additional 51 sheep were matched by breed and farm with other animals in the OvineHD dataset, and these additional animals were genotyped with the Illumina OvineSNP50 array. All unphased genotypes were converted from the. ped format of PLINK v1.9 (Purcell et al., 2007; Chang et al., 2015) to variant call format using a script incorporating the data. table v1.11.4 package in R v3.3.2 (R Core Team, 2016). Before imputation, loci without available position information and loci with a call rate lower than 95% from either array were removed using a script incorporating the same R package. Finally, a similar R script was used to reassemble the dataset following group-specific imputation (for groups consisting of Katahdin sheep, sheep with Barbados Blackbelly heritage, and other breeds to represent the three largest genetic groupings in this dataset). For sheep genotyped with the OvineHD BeadChip, genotype information was added for loci not already present in the OvineSNP50 dataset, and this group was designated as a reference panel. In this reference panel, Beagle v5.0 was used to impute sporadic missing genotypes and then to phase the imputed genotypes (Browning and Browning, 2007) with default settings (Browning et al., 2018). Using the Beagle v5.0 default settings, only the sheep genotyped on the OvineSNP50 BeadChip were imputed to the OvineHD BeadChip marker set and phased (Browning and Browning, 2007; Browning et al., 2018).

### 2.3 Statistical analysis

Genome-wide association was performed in SNP and Variation Suite (SVS) version 8 (Golden Helix, Inc., Bozeman, MT, US) (Lee et al., 2012). Initial quality control was performed to remove variants with minor allele frequency below 2% and Hardy-Weinberg equilibrium tests with *p* < 10^–25^. Initial association models were constructed in the EMMAX (Kang et al., 2010) implementation within SVS containing fixed effects of breed, farm, and principal components derived from genome-wide genotypes (Price et al., 2006; Zhang and Pan, 2015) as well as a random term for the genomic relationship matrix. However, the breed term was multi-colinear with the principal components, indicating that the principal components contained the same information as the breed term. Indeed, preliminary analysis showed *R* ^2^ between breed and the first principal component alone was 0.679, *R* ^2^ between breed and the first two principal components was 0.949, and *R* ^2^ between breed and all 20 principal components was >0.999 so the breed term was dropped in favor of the principal components. Final association models were mixed models including fixed effects for the additive contribution of the SNP of interest, farm, and 20 principal components derived from genome-wide genotypes, as well as the genomic relationship matrix as a random effect. Genome-wide significance was determined by *p* < 5 × 10^−7^, and genome-wide suggestive evidence was determined by *p* < 1 × 10^−5^ (Burton et al., 2007). Manhattan plots and Q-Q plots were constructed in R using the mhplot2 script kindly provided by Dr. Stephen Turner (http://gettinggeneticsdone.blogspot.com/2011/04/annotated-manhattan-plots-and-qq-plots.html, viewed on 11-15-11).

## 3 Results

There were 92 sheep with healthy scores of 1 (no footrot in any foot), 52 with intermediate scores of 2 (at least one foot with a score of 2), and 107 with scores of 3 for footrot disease (at least one foot with a score of 3). Out of 553,197 SNPs, the call rate screen removed no SNPs with call rates <95%. The minor allele frequency screen removed 37,043 SNPs, and the Hardy-Weinberg equilibrium test at *p* < 10^–25^ removed an additional 519 SNPs, which left 515,635 SNPs after quality control for further analysis.

**FIGURE 1:**
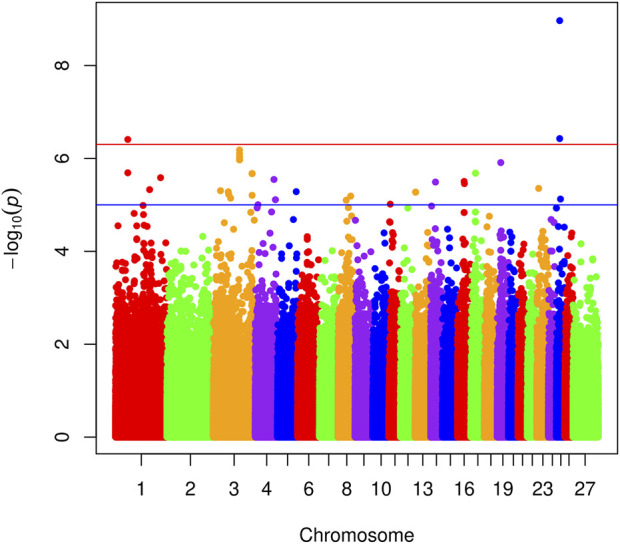
Manhattan plots showing SNP association with footrot. Different colors indicate various ovine chromosomes. The *x*-axis shows SNP position across chromosomes in numerical order, and the *y*-axis represents the −log10 (*p*-values). The upper and lower lines indicate the genome-wide significant and suggestive thresholds, respectively.

The genomic inflation factor (lambda) for the overall genome-wide association analysis was 1.01. Figure 1 shows a Manhattan plot of genome-wide association, and a quantile-quantile plot is given in Supplementary Figure S2. Detailed information on the top loci demonstrating genome-wide significant associations are highlighted in Table 1. Additional information on loci with genome-wide suggestive association are shown in Table 2.

**TABLE 1:** Genome-wide significant single nucleotide polymorphism (SNP) markers associated with footrot.

Chr	refSNP	Variant type	Position bp	A1	A2	MAF	*p*-value	Genes within 100 Kb
1	*rs159679616*	missense variant	66,009,064	C^+^	G	0.024	3.91 × 10^−7^	Glogin subfamily A member 6-like protein 22 (LOC101114579), Guanylate-binding protein 6-like (*GBP6*)
25	*rs421352693*	intergenic variant	18,039,266	T^+^	C	0.024	1.08 × 10^−9^	No close gene or protein coding sequence
25	*rs411314769*	intergenic variant	18,084,729	A^+^	G	0.038	3.74 × 10^−7^	No close gene or protein coding sequence

+ represents the favorable host allele against footrot.

**TABLE 2:** Genome-wide suggestive single nucleotide polymorphism (SNP) markers associated with footrot.

Chr	refSNP	Variant type	Position bp	A1	A2	MAF	*p*-value	Genes within 100 Kb
1	*rs159679642*	downstream gene variant	66,005,263	C^+^	T	0.052	2.04 × 10^−6^	Glogin subfamily A member 6-like protein 22 (LOC101114579), Guanylate-binding protein 6-like (*GBP6*)
1	*rs430349561*	intergenic variant	182,882,844	A	G^+^	0.032	4.70 × 10^−6^	MYCBP associated and testis expressed 1 (*MAATS1*), Nuclear receptor subfamily 1 group I member 2 (*NR1I2*)
1	*rs429765562*	intergenic variant	241,551,968	T^+^	C	0.039	2.60 × 10^−6^	No close gene or protein coding sequence
3	*rs427476105*	intergenic variant	140,124,049	T^+^	G	0.024	1.07 × 10^−6^	No close gene or protein coding sequence
3	*rs430672094*	intergenic variant	140,130,660	T^+^	C	0.024	1.07 × 10^−6^	No close gene or protein coding sequence
3	*rs417462455*	intergenic variant	140,150,750	C^+^	T	0.024	1.07 × 10^−6^	No close gene or protein coding sequence
3	*rs415897197*	intergenic variant	140,123,914	A^+^	G	0.026	7.89 × 10^−7^	No close gene or protein coding sequence
3	*rs415053617*	intergenic variant	140,073,084	G^+^	A	0.024	6.60 × 10^−7^	Solute carrier family 38 member 2 (*SLC38A2*)
3	*rs426283825*	intergenic variant	140,173,114	A^+^	G	0.029	9.35 × 10^−7^	No close gene or protein coding sequence
3	*rs421757376*	intergenic variant	38,174,475	T^+^	C	0.028	4.96 × 10^−6^	Poly(rC) binding protein 1 (*PCBP1*)
3	*rs409808538*	intron variant	207,436,102	T	G^+^	0.041	2.12 × 10^−6^	Lysophosphatidylcholine acyltransferase 3 (*LPCAT3*), EMG1 N1-specific pseudouridine methyltransferase (*EMG1*), Prohibitin 2 (*PHB2*), Protein tyrosine phosphatase non-receptor type 6 (*PTPN6*), Chromosome 3 C12orf57 homolog (*C3H12orf57*), Atrophin 1 (*ATN1*), Enolase 2 (*ENO2*)
3	*rs159823349*	synonymous variant	207,439,936	G^+^	A	0.042	2.12 × 10^−6^	Lysophosphatidylcholine acyltransferase 3 (*LPCAT3*), EMG1 N1-specific pseudouridine methyltransferase (*EMG1*), Prohibitin 2 (*PHB2*), Protein tyrosine phosphatase non-receptor type 6 (*PTPN6*), Chromosome 3 C12orf57 homolog (*C3H12orf57*), Atrophin 1 (*ATN1*), Enolase 2 (*ENO2*)
3	*rs430419641*	-	-	T^+^	G	0.042	2.12 × 10^−6^	Not mapped to the genome
3	*rs414749931*	intergenic variant	79,863,459	G^+^	A	0.032	5.24 × 10^−6^	Prolyl endopeptidase like (*PREPL*)
3	*rs426897991*	intergenic variant	82,276,667	A	G^+^	0.033	5.96 × 10^−6^	No close gene or protein coding sequence
3	*rs161809555*	missense variant	207,542,450	G	A^+^	0.051	6.22 × 10^−6^	Enolase 2 (*ENO2*), Leucine rich repeat containing 23 (*LRRC23*), Triosephosphate (*TPI1*), Ubiquitin specific peptidase 5 (*USP5*), Cell division cycle associated 3 (*CDCA3*), G protein subunit beta 3 (*GNB3*), Prolyl 3-hydroxylase 3 (*P3H3*), G protein-coupled receptor 162 (*GPR162*), CD4 molecule (*CD4*)
3	*rs424145176*	intergenic variant	92,413,143	C^+^	T	0.081	7.17 × 10^−6^	Transforming protein RhoA (LOC101106246), Transforming growth factor alpha (*TGFA*)
4	*rs416121047*	intergenic variant	100,351,736	C^+^	G	0.032	2.84 × 10^−6^	No close gene or protein coding sequence
4	*rs420577155*	intergenic variant	108,031,285	A^+^	G	0.043	7.74 × 10^−6^	No close gene or protein coding sequence
4	*rs418147929*	intergenic variant	14,801,187	T^+^	C	0.030	9.95 × 10^−6^	No close gene or protein coding sequence
5	*rs428564305*	intergenic variant	100,414,308	A^+^	G	0.080	5.20 × 10^−6^	No close gene or protein coding sequence
8	*rs422048023*	intergenic variant	43,382,115	G^+^	A	0.029	7.95 × 10^−6^	EPH receptor A7 (*EPHA7*)
8	*rs399612094*	3 prime UTR variant	67,232,813	T^+^	C	0.073	6.48 × 10^−6^	ENSOARG0000002740
11	*rs427616272*	intergenic variant	7,537,149	T^+^	C	0.039	9.66 × 10^−6^	A-kinase anchoring protein 1 (*AKAP1*)
13	*rs419736982*	intron variant	2,021,618	G	A^+^	0.025	5.21 × 10^−7^	Phospholipase C beta 4 (*PLCB4*), Lysosomal associated membrane protein family member 5 (*LAMP5*), p21(*RAC1*) activated kinase 5 (*PAK5*)
13	*rs429709544*	synonymous variant	2,045,662	A	G^+^	0.025	5.33 × 10^−6^	Lysosomal associated membrane protein family member 5 (*LAMP5*), p21 (*RAC1*) activated kinase 5 (*PAK5*)
14	*rs417508179*	intron variant	25,429,390	G^+^	A	0.045	3.23 × 10^−6^	Matrix metallopeptidase 15 (*MMP15*), Cilia and flagella associated protein 20 (*CFAP20*), Casein kinase 2 alpha 2 (*CSNK2A2*)
16	*rs409491906*	downstream gene variant	35,417,010	C	A^+^	0.055	3.13 × 10^−6^	RPTOR independent companion of MTOR complex 2 (*RICTOR*), Oncostatin M receptor (*OSMR*)
16	*rs411963224*	intron variant	36,857,015	A^+^	G	0.033	3.49 × 10^−6^	WD repeat domain 70 (*WDR70*), Nuceloporin 155 (*NUP155*)
17	*rs426749853*	intergenic variant	24,462,154	A	G^+^	0.021	2.08 × 10^−6^	No close gene or protein coding sequence
19	*rs424837077*	intron variant	19,106,626	T^+^	C	0.39	1.23 × 10^−6^	Glutamate metabotropic receptor 7 (*GRM7*)
23	*rs398222764*	intergenic variant	9,724,724	G^+^	A	0.025	4.42 × 10^−6^	No close gene or protein coding sequence
25	*rs409708600*	intron variant	22,379,923	A^+^	G	0.039	7.50 × 10^−6^	Catenin alpha 3 (*CTNNA3*)

+ represents the favorable host allele against footrot.

## 4 Discussion

Since footrot susceptibility is heritable (Parker et al., 1983; Raadsma et al., 1994; Nieuwhof et al., 2008b; Raadsma and Conington, 2011), a genetic selection approach may help to decrease lesion score and the number of animals affected through selective breeding if loci associated with footrot can be identified. The aim of the present study was to perform the first GWAS for footrot in North American wool sheep and also the first GWAS for hair sheep. Our analysis yielded an appropriate model fit with a genomic inflation factor of 1.01. We identified 3 genome-wide significant and 33 genome-wide suggestive loci associated with ovine footrot susceptibility on 13 different autosomes (Table 1; Table 2). Below, we explore the regions surrounding these regions, the potential involvement of nearby genes in ovine footrot, and compare our results to those identified in other studies to date.

Multiple genome-wide significant positional candidate genes were identified in the present study with functions in the immune system (Table 1). First, two genome-wide associate SNPs (*rs421352693*; *p* = 1.08 × 10^−9^ and *rs411314769*; *p* = 3.74 × 10^−7^; Table 1) were located in an intergenic region on OAR25. Although this locus did not contain a positional candidate gene, this region could affect susceptibility to footrot through the regulation of distant genes due to the presence of regulatory elements, non-coding RNAs, or other features (Scacheri and Scacheri, 2015; Georges et al., 2019). It is interesting that the most significant SNP (*rs421352693*) was located less than 10 Kb away from a seven base-pair highly conserved element from an analysis of 91 eutherian mammal genomes (Ensembl release 97; Zerbino et al., 2018). The specific function of this highly conserved element has not been fully elucidated, but it is located between *ARID5B* and *RTKN2* (Jiang et al., 2014), both of which have been linked to roles in regulation of immune responses. *ARID5B* encodes a DNA-binding protein with roles in NK cell function (Cichocki et al., 2018), cancers of both B and T lymphocytes (Healy et al., 2010; Zeng et al., 2014; Leong et al., 2017), and autoimmune disease (Wang et al., 2012; Yang et al., 2013). The *RTKN2* gene is expressed in lymphocytes (Collier et al., 2004), induces an NF-kB-dependent hold on apoptosis (Collier et al., 2009) that can change counts and function of available immune cells, and has been implicated in autoimmune disease (Myouzen et al., 2012). In the cases of both of these genes, roles in immune cells suggest possible immune mechanisms for differential control of footrot in sheep. There are ongoing efforts to identify and annotate regulatory elements in sheep and other ruminants through the Functional Annotation Animal Genomes (FAANG) consortium, among others (Andersson et al., 2015; Tuggle et al., 2016). The functional importance of this genome-wide association may become clearer once data from such annotation projects are complete.

The guanylate-binding protein 6 (*GBP6*) gene is located on OAR1 (Jiang et al., 2014) within a peak defined by two SNPs, one genome-wide significant (rs159679616; *p* = 3.91 × 10^−7^; Table 1) and one genome-wide suggestive (*rs159679642*; *p* = 1.02 × 10^−6^; Table 2). Guanylate-binding proteins (GBPs) are abundantly expressed cellular proteins with seven highly homologous members in sheep, termed GBP1 to GBP7, expressed in response to interferon-gamma (IFN-γ) and other pro-inflammatory cytokines (Olszewski et al., 2006; Kim et al., 2011; Praefcke, 2018). *GBP6* stimulates phagocyte oxidase, antimicrobial peptides, and autophagy effectors in an immune response capable of killing multiple types of bacteria (Kim et al., 2011). Furthermore, it has been proposed that GBPs might promote lysis of vacuoles, thereby triggering detection of pathogen associated molecular patterns (PAMPs) and further immune responses, as well (Meunier et al., 2014; Meunier and Broz, 2015).

The SNP association peak on OAR1 was also very near the trichohyalin (*TCHH*) gene (Table 1; Table 2). Trichohyalin crosslinks with keratin intermediate filaments to provide mechanical strength in hair follicles, as well as hooves (O’Keefe et al., 1993; Steinert et al., 2003). This could affect hoof formation in ways that predispose or protect sheep from ovine footrot. Thus, this most significant pair of SNPs result points to involvement of hoof structure as well as to stimulation of the immune response as potential mechanisms for susceptibility to ovine footrot. Other immune related QTLs have been reported in the literature from the same region where *GBP6* and *TCHH* are located on OAR1. For instance, a facial eczema susceptibility QTL has been reported based on microsatellite marker genotyping on OAR1 (Phua et al., 2009). It is possible that this association could reflect contributions from both genes (*GBP6* and *TCHH*) through one or more regulatory elements, but future work would be required to elucidate the specific underlying functional mutation(s) in this region.

A cluster of six SNPs spanning just over 100 Kb on OAR3 included the lowest *p*-value genome-wide suggestive association results (Table 2). Of these, the SNP with the lowest *p*-value (*p* = 6.6 × 10^−7^) was rs415053617 (Table 2), which was located less than 10 Kb from *SLC38A1* (Oguchi et al., 2012). The *SLC38A1* gene encodes a glutamine transporter expressed in hair cells (Oguchi et al., 2012) and extracellular exosomes (Willms et al., 2016) and is involved in oxidative stress responses (Ogura et al., 2011). Taken together, these data suggest *SLC38A1* might inhibit *D. nodosus* and/or *F. necrophorum* by limiting glutamine nutrient availability, either before or during infection.

Two GWAS have been performed previously for ovine footrot susceptibility in sheep with wool. Niggeler et al. (2017) identified the only other genome-wide association with footrot (aside from the present work) on OAR2 near the multi-PDZ domain protein 1 (*MPDZ*) gene in Swiss White Alpine sheep. The genome-wide associated SNP was rs418747104, and additional genome-wide suggestive markers nearby included *rs426927857* and *rs406749947*. However, this locus was not associated with footrot in our dataset (all *p* > 0.05). The other prior GWAS for ovine footrot in sheep with wool (Mucha et al., 2015) identified no genome-wide associations but did identify chromosome-wise significant associations on ovine chromosomes 4, 8, 14, 17, 18, 24, and 26. None of these loci were associated with footrot in this study (all *p* > 0.05). The non-overlapping associated genomic regions among different experiments may be caused by differences in pathogen characteristics on different continents or by breed differences, especially since this is the first study to include hair sheep.

In addition to GWAS, prior candidate gene studies have been conducted to identify associations with ovine footrot. Candidate gene studies have identified associations with footrot in *DQA1*, *DQA2*, *DQB*, and *DRA* in the Major Histocompatibility Complex (MHC) on ovine chromosome 20 (Escayg et al., 1997; Gelasakis et al., 2013). This is a complex region with paralogs derived from tandem repeats and even variable numbers of genes (Herrmann-Hoesing et al., 2008; Gao et al., 2010; Bickhart and Liu, 2014). Haplotypes in this region can be quite long, and some are ancient in origin (Raymond et al., 2005). The specific markers used in prior work are difficult to assess in our study because of the widely differing marker types. However, no markers in that region on OAR 20 achieved genome-wide significant or genome-wide suggestive support in this study (Table 1; Table 2).

## 5 Conclusion

This is the first genome-wide study examining footrot susceptibility using hair sheep and the first GWAS to identify multiple genome-wide associations with footrot. These results provide insight into mechanisms that may affect footrot susceptibility and resistance. In particular, both genome-wide significant and genome-wide suggestive associations illustrated themes of immune function, nutrient availability, and hoof formation and integrity. Thus, this study met its objective in improving understanding of host genetics of footrot susceptibility. In addition to biological insights, these results provide a foundation for future work developing predictive genetic marker tests. Such technology offers the possibility of disproportionate benefits to flock health through selection against the most susceptible animals before infection, disease, and transmission to other animals (Galvani and May 2005; Lloyd-Smith et al., 2005). However, more research is needed to identify the specific functional mutations in linkage disequilibrium with the markers in this study. In addition, the functional mutations will need to be validated and examined for potential correlated responses to selection, including production traits (White and Knowles, 2013) such as some already identified for immune loci in sheep (Cinar et al., 2016; Cinar et al., 2018). Further, additional animals can be used to examine potential for genomic selection to leverage these data into additional breeding applications that may not require identification of causal mutations. In these ways, sheep production can benefit from reliable, predictive genetic tests for which selection does not lead to deleterious effects on other traits.

## Data Availability

The datasets presented in this study can be found in online repositories. The names of the repository/repositories and accession number(s) can be found below: https://osf.io/, DOI 10.17605/OSF.IO/FX3ESat.
