# Supplementary figures and images for: Genome-wide association with footrot in hair and wool sheep
Source: Front Genet. 2024 Jan 15;14:1297444. doi: 10.3389/fgene.2023.1297444 (PMC10822918; doi:10.3389/fgene.2023.1297444)

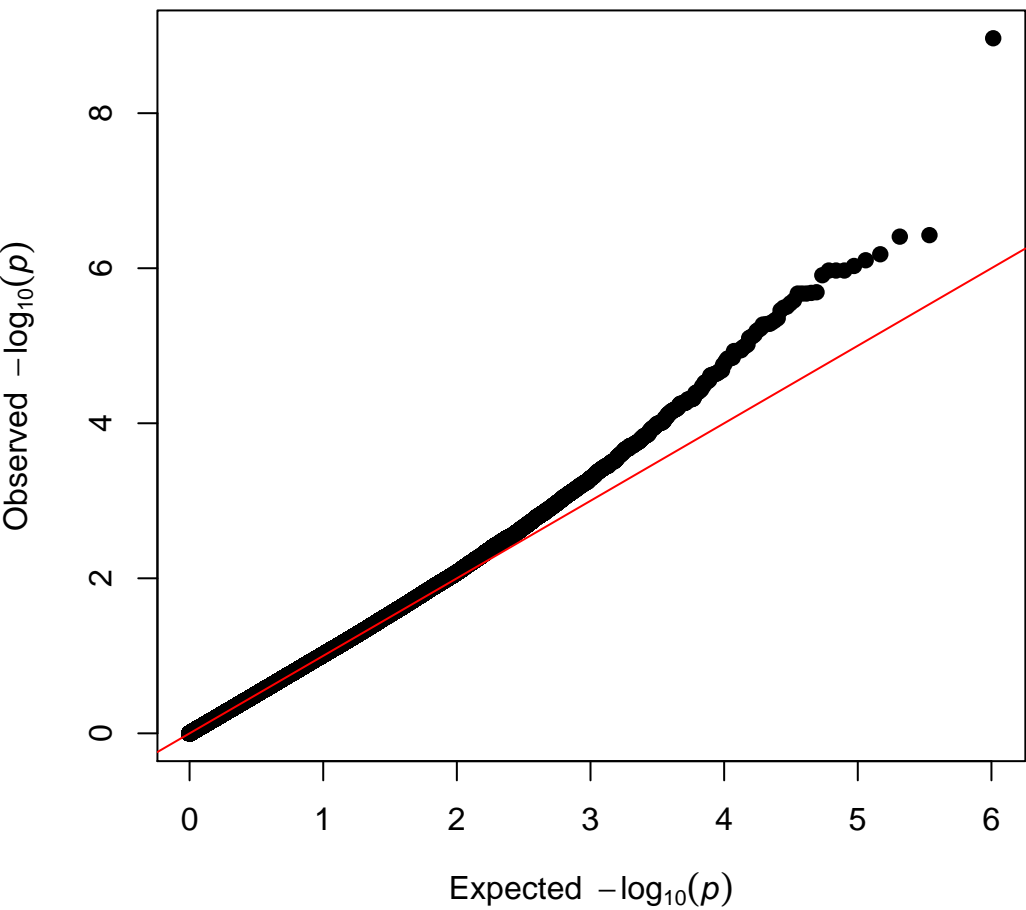

Supplement: Supplementary file 1 [file DataSheet2.pdf]
