# Supplementary material for: Genome-wide association with footrot in hair and wool sheep
Source: Front Genet. 2024 Jan 15;14:1297444. doi: 10.3389/fgene.2023.1297444 (PMC10822918; doi:10.3389/fgene.2023.1297444)

Figure S1. Sample hoof scores

1. A score of 1 is a “clean hoof” showing no signs of infection or inflammation.

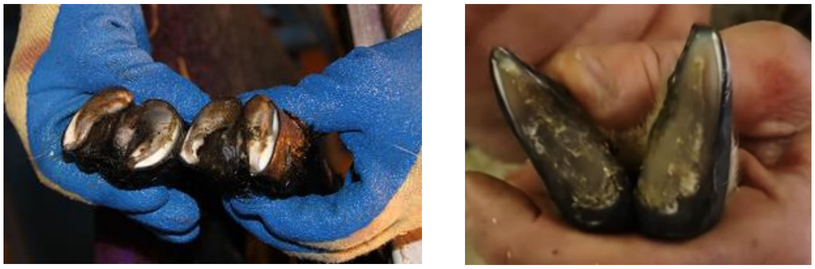

2. A score of 2 on a hoof designates uncertainty. No rot is evident by sight or smell of these hooves, but signs or symptoms of inflammation, swelling, lameness, or soreness might be detected.

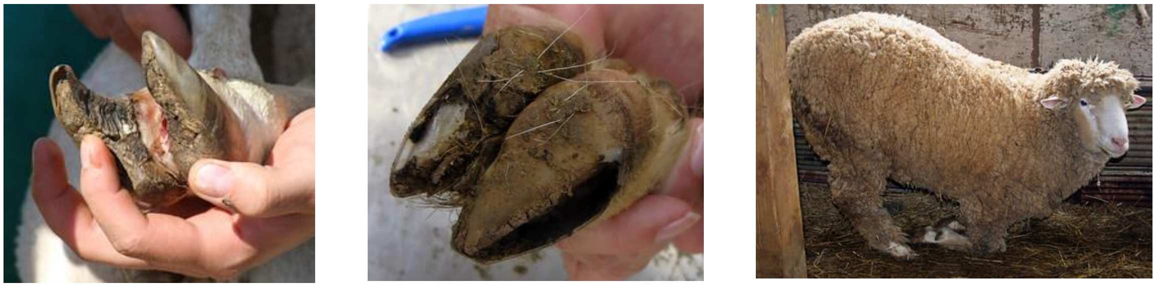

3. Any hoof that has rotting tissue on any part of the hoof is scored as a 3. There will be a putrid smell to the feet of sheep with foot rot.

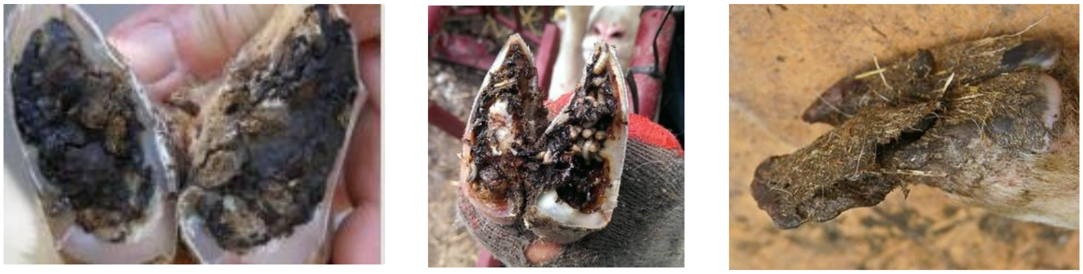

Supplement: Supplementary file 2 [file DataSheet1.pdf]
